# The clinical association of programmed cell death protein 4 (PDCD4) with solid tumors and its prognostic significance: a meta-analysis

**DOI:** 10.1186/s40880-016-0158-3

**Published:** 2016-11-16

**Authors:** John Zeng Hong Li, Wei Gao, Wai-Kuen Ho, Wen Bin Lei, William Ignace Wei, Jimmy Yu-Wai Chan, Thian-Sze Wong

**Affiliations:** 1 Department of Surgery, The University of Hong Kong, Queen Mary Hospital, Hong Kong, Hong Kong, SAR P. R. China; 2 Department of Otolaryngology, The First Affiliated Hospital of Sun Yat-sen University, Guangzhou, Guangdong 510080 P. R. China

**Keywords:** Programmed cell death protein 4 (PDCD4), Solid tumor, Meta-analysis, Prognosis, Overall survival, Disease-free survival, Recurrence-free survival

## Abstract

**Background:**

Programmed cell death protein 4 (PDCD4) is a novel tumor suppressor protein involved in programmed cell death. Its association with cancer progression has been observed in multiple tumor models, but evidence supporting its association with solid tumors in humans remains controversial. This study aimed to determine the clinical significance and prognostic value of PDCD4 in solid tumors.

**Methods:**

A systematic literature review was performed to retrieve publications with available clinical information and survival data. The eligibility of the selected articles was based on the criteria of the Dutch Cochrane Centre proposed by the Meta-analysis Of Observational Studies in Epidemiology group. Pooled odds ratios (ORs), hazard ratios (HRs), and 95% confidence intervals (CIs) for survival analysis were calculated. Publication bias was examined by Begg’s and Egger’s tests.

**Results:**

Clinical data of 2227 cancer patients with solid tumors from 23 studies were evaluated. PDCD4 expression was significantly associated with the differentiation status of head and neck cancer (OR 4.25, 95% CI 1.87–9.66) and digestive system cancer (OR 2.87, 95% CI 1.84–4.48). Down-regulation of PDCD4 was significantly associated with short overall survival of patients with head and neck (HR: 3.44, 95% CI 2.38–4.98), breast (HR: 1.86, 95% CI 1.36–2.54), digestive system (HR: 2.12, 95% CI 1.75–2.56), and urinary system cancers (HR: 3.16, 95% CI 1.06–9.41).

**Conclusions:**

The current evidence suggests that PDCD4 down-regulation is involved in the progression of several types of solid tumor and is a potential marker for solid tumor prognoses. Its clinical usefulness should be confirmed by large-scale prospective studies.

## Background

Programmed cell death protein 4 (PDCD4) is a novel tumor suppressor protein involved in programmed cell death. The *PDCD4* gene is located on chromosome 10q24, and its allelic loss/gain is frequently reported in human cancers. Up-regulation of PDCD4 is observed after the initiation of apoptosis, suggesting that loss of, or reduced, *PDCD4* expression could contribute to the anti-apoptotic property of cancer cells [1]. In mouse epidermal JB6 cells, which are resistant to anchorage-dependent cell death and neoplastic transformation, high levels of *PDCD4* expression could be induced in response to the presence of tumor promoters such as 12-O-tetradecanoylphorbol-13-acetate [2] and tumor necrosis factor-alpha [3]. Therefore, it has been suggested that *PDCD4* is a potent tumor suppressor gene. PDCD4 could inhibit neoplastic transformation through inhibition of adaptor protein-1 (AP-1) activation [3]. Structurally, PDCD4 could interact with RNA helicase eukaryotic translation initiation factor 4A (eIF4A), inhibiting its helicase activity and affecting protein translation [4, 5]. In addition, PDCD4 could inhibit nuclear factor kappaB (NF-κB)-dependent transcription and related pathways [6].

Loss of important tumor suppressors with critical functions during the transformation process is a hallmark of cancer. Identifying key tumor suppressor proteins is important for the sub-classification of tumors at different stages with different behaviors. Moreover, the elucidation of the pathways associated with tumor suppressors could help identify predictive markers for prognostic use and provide novel insights into cancer treatment. Accumulated results in preclinical studies indicate that *PDCD4* is a novel tumor suppressor gene with anti-neoplastic properties [7–9]. Nevertheless, some studies have suggested the conflicting conclusion that PDCD4 does not exert a tumor-suppressing effect in certain malignancies, such as non-small cell lung cancer [10, 11]. To explore whether PDCD4 consistently acts as a tumor suppressor and positive prognostic marker for solid tumors, we conducted an updated meta-analysis to evaluate the clinical significance and prognostic value of PDCD4 in human cancers.

## Methods

### Literature search

A systematic literature search through PubMed, EMBASE, and MEDLINE was performed using the following main keywords: “PDCD4” and “cancer” or “carcinoma” or “tumor” or “malignancy.” All studies that examined the expression status of PDCD4 were recruited regardless of the detection methods used. The last search was performed on January 12th, 2016.

### Study selection

Two reviewers (JZHL and WG) manually screened and selected the eligible studies independently. Studies that were not reported in English or Chinese, review articles, studies that had used cell lines and animal models without any data on human tissue samples, and studies without sufficient data for calculation were excluded from the analysis.

### Methodology quality assessment

The studies on prognosis were evaluated using the criteria of the Dutch Cochrane Centre proposed by Meta-analysis Of Observational Studies in Epidemiology (MOOSE) group [12]. The following inclusion criteria were used: (1) trial dealt with PDCD4; (2) clear definition of study design; (3) clear definition of outcome assessment, including overall survival (OS), disease-specific survival (DSS), disease-free survival (DFS), and relapse-free survival (RFS); (4) clear definition of cut-off score of PDCD4 expression or high/low evaluation; and (5) a follow-up period of at least 12 months.

### Data extraction

The selected publications were accessed by two reviewers (JZHL and WG). The following details were retrieved from the selected papers: (1) general information, including the first author, publication year, case populations, cancer types, sample types, and test methods; (2) the case number in each of the sub-groups with different PDCD4 expression levels and diverse clinicopathologic properties; and (3) the results of survival analysis, including hazard ratios (HRs) and 95% confidence intervals (CIs).

### Statistical analysis

Pooled odd ratios (ORs), HRs, and 95% CIs were calculated for the evaluation of the clinicopathologic association of PDCD4 and its prognostic value in solid tumors. To check whether there was homogeneity among the individual ORs/HRs for the selection of the optimal effects model analysis, a heterogeneity test with the inconsistency index (*I* ^*2*^) statistic and *Q* statistic (*P* value) was performed. Substantial heterogeneity was indicated when *I* ^*2*^ ≥ 50% and *P* < 0.05, and a random effects model was adopted; a fixed effects model was appropriate when *I* ^*2*^ < 50% and *P* > 0.05. After a suitable model had been chosen, Forest plots with pooled OR/HR and 95% CIs were then retrieved from the individual HRs and 95% CIs. Pooled OR/HR > 1 suggested that the worst prognoses were more likely to occur in the patients with no, or low levels of, PDCD4 expression than those with high levels of PDCD4 expression. Statistically significant differences between groups with diverse PDCD4 expression levels was determined if the 95% CI did not overlap 1. In addition to the calculation of overall OR/HR and 95% CI, subgroup analysis was performed with respect to the case population (Asian/European/North American), cancer type (brain tumor/head and neck cancer/breast cancer/digestive system cancer/respiratory system tumor/gynecologic tumor/urinary system cancer), and sample type (protein/RNA).

Publication bias was assessed by the Begg’s and Egger’s tests [13]. *P* < 0.05 represented a statistically significant publication bias. All analyses were performed with Stata software version 12.0 (Stata Corporation, College Station, TX, USA).

## Results

### Study characteristics and qualitative assessment

**Fig. 1:**
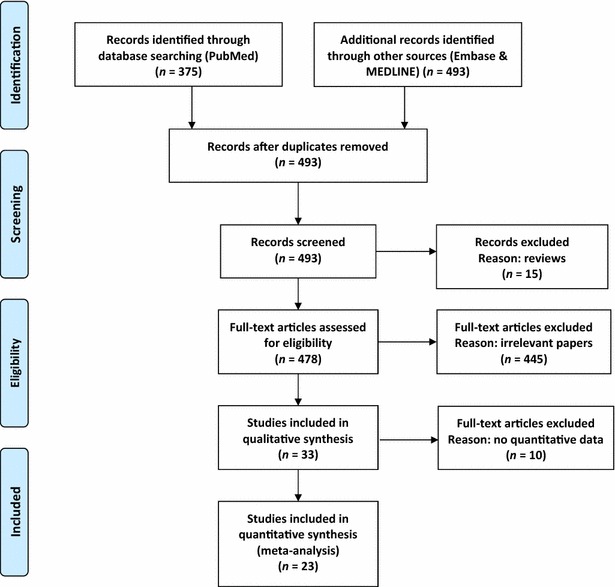
Preferred reporting items for systematic reviews and meta-analyses (PRISMA) flow diagram showing the literature search strategy and review process. In total, 493 articles were found from three databases; after multiple-step selection, 23 eligible articles were finally included in the meta-analysis

According to the selection criteria, 493 articles were found in the initial screening. After removing 445 irrelevant articles, 33 articles were selected for further evaluation. According to the critical checklist of the Dutch Cochrane Centre, 23 articles fulfilled all of the quality assessment criteria. These 23 articles, involving a total of 2227 solid tumor cases, were included in the meta-analysis. Figure 1 shows the selection process. The characteristics of the included studies are shown in Table 1.

**Table 1:** Detailed characteristics of the 23 studies included in the meta-analysis

Publication year	Author	Nationality	Total cases	Cancer type	Therapy	Follow-up (months)	PDCD4 expression (cases)	Protein location	Examine methods	Dichotomic criteria	Cut-off value	Study variables
2003	Chen et al. [22]	Germany	124	Lung cancer	NA	24 (2–66)	21	NA	IHC	Positive/negative	IHC score = 1	N category, clinical stage, histological differentiation
2007	Mudduluru et al. [28]	Germany	71	Colorectal cancer	Surgery	36 (1–72)	13	Nucleus and cytoplasm	IHC	Positive/negative	IHC score = 1	OS, DSS
2008	Wang et al. [34]	China	43	Ovarian epithelial carcinoma	NA	50 (NA)	18	Nucleus and cytoplasm	IHC	Strong, moderate, weak, negative	IHC score = 3	Clinical stage, DSS, histological differentiation
2009	Gao et al. [33]	China	84	Glioma	Surgery	36 (NA)	16	NA	IHC	Positive/negative	Positive (+)	Clinical stage, DSS
	Wei et al. [35]	China	79	Ovarian invasive ductal carcinoma	Surgery + chemotherapy	49 (26–97)	40	Total	Western blotting	High/low	Median	DFS
2010	Motoyama et al. [23]	Japan	105	Gastric cancer	NA	30 (1.2–134.4)	28	NA	QPCR	High/low	T/N ratio = 1	N category, M category, clinical stage, histological differentiation
	Reis et al. [29]	Canada	50	Oral cancer	NA	108 (NA)	25	NA	qPCR	High/low	Median	OS, DFS
2011	Lim et al. [19]	Korea	108	Colorectal cancer	NA	NA	64	NA	IHC	Strong, moderate, weak, negative	IHC score = 2	T category, N category, M category, clinical stage, histological differentiation
	Feng et al. [20]	China	54	Laryngeal cancer	NA	NA	33	Nucleus and cytoplasm	IHC	Positive/negative	IHC score = 4	N category, clinical stage, histological differentiation
	Kakimoto et al. [50]	Japan	19	Gastric cancer	NA	NA	6	Nucleus or cytoplasm	IHC	Strong, moderate, weak, negative	IHC score = 3	Histological differentiation
2012	Ding et al. [14]	China	63	Gastro-intestinal stromal tumor	NA	NA	21	NA	IHC	High/low	IHC score = 4	Tumor size
	Nagao et al. [15]	Japan	65	Pancreatic ductal adenocarcinoma	NA	NA	24	Nucleus or cytoplasm	IHC	Strong, moderate, weak, negative	30% cancer cells with PDCD4 (+)	Tumor size, T category, N category, OS, histological differentiation
	Cao et al. [16]	Korea	205	Gastric cancer	NA	NA	136	NA	IHC	Positive/negative	5% cancer cells with PDCD4 (+)	Tumor size
	Li et al. [17]	China	66	Renal cell carcinoma	Surgery	47.3 (18–65)	31	Nucleus & cytoplasm	IHC	High/low	IHC score = 2	Tumor size, T category, M category, OS, histological differentiation
	Wang et al. [21]	China	60	Laryngeal cancer	NA	NA	26	NA	IHC	Positive/negative	30% cancer cells with PDCD4 (+)	N category, clinical stage, histological differentiation
	Meric-Bernstam et al. [30]	USA	188	Breast cancer	Surgery + endocrine therapy	87 (1–197)	78	NA	IHC	Positive/negative	5% cancer cells with PDCD4 (+)	Tumor size
	Horiuchi et al. [31]	Japan	118	Colorectal cancer	NA	45 (2–89)	61	NA	qPCR	High/low	T/N ratio = 1	OS, DFS
2013	Guo et al. [7]	China	122	Gastric cancer	NA	42 (3–84)	36	NA	IHC	Positive/negative	6% cancer cells with PDCD4 (+)	Tumor size, N category, OS
	Zhen et al. [18]	China	190	Naso-pharyngeal cancer	NA	NA (4–126)	73	Cytoplasm	IHC	High/low	IHC score = 5	T category, N category, M category, clinical stage, OS
	Qi et al. [24]	China	96	Salivary adenoid cystic carcinoma	Surgery	42 (3–68)	34	Nucleus and cytoplasm	IHC	High/low	IHC score = 4	Clinical stage, OS, DSS
	Ma et al. [26]	China	195	Digestive system cancer	NA	NA	93	Cytoplasm	IHC	Positive/negative	30% cancer cells with PDCD4 (+)	Clinical stage, histological differentiation
2014	Yu et al. [27]	China	30	Gastric cancer	NA	NA	11	NA	IHC	High/low	Stain index score = 6	Histological differentiation
	Dou et al. [32]	China	92	Advanced rectal cancer	Surgery + nCRT	60 (NA)	40	Nucleus and cytoplasm	IHC	High/low	30% cancer cells with PDCD4 (+)	OS, DFS

In total, 2227 solid tumor cases from 23 studies were included in the meta-analysis. The prognostic value of programmed cell death protein 4 (PDCD4) expression in multiple solid tumors was studied, and stratified analysis was performed according to nationality, cancer type, and sample type. The follow-up data are presented as median with range in parentheses

*NA* not available (not mentioned), *IHC* immunohistochemistry, *OS* overall survival, *DSS* disease-specific survival, *DFS* disease-free survival, *RFS* recurrence-free survival, *qPCR* quantitative polymerase chain reaction, *nCRT* neoadjuvant chemoradiotherapy

### Associations of PDCD4 down-regulation with the clinicopathologic parameters of cancer patients

The associations between PDCD4 expression and the clinicopathologic features of patients with solid tumors are shown in Table 2. Four studies of digestive system cancers [7, 14–16] and one study of urinary system cancers [17] examined associations between tumor size and PDCD4 expression level. All of the studies were carried out on Asian patients using antigen-based methods, and the combined OR indicated no significant association. Low PDCD4 expression level was associated with advanced T category of urinary system cancers (OR 4.87, 95% CI 1.69–14.00) [17] and head and neck cancers (OR 2.15, 95% CI 1.10–4.19) [18]. However, PDCD4 expression level was not associated with the T category of digestive system cancers (OR 0.98, 95% CI 0.46–2.08) [15, 19]. There was no obvious evidence for an association between PDCD4 expression and the N category of head and neck cancers [20, 21], respiratory system cancers [22], and digestive system cancers [7, 15, 19, 23]. Low PDCD4 level was associated with advanced M category of urinary system cancers (OR 4.87, 95% CI 1.69–14.00) [17] and advanced clinical stage of head and neck cancers (OR 2.30, 95% CI 1.44–3.69) [18, 20, 21, 24].

**Table 2:** Associations between PDCD4 down-regulation and the clinicopathologic parameters of patients with solid tumors

Parameter	Study subjects	Reference(s)	Total cases	PDCD4 expression (cases)^a^		OR	95% CI	*I* ^*2*^ (%)	*P*
				Negative	Positive				
Tumor size (large vs. small)	Tumor type								
	Digestive system cancer	[7, 14–16]	530	114 vs. 169	84 vs. 163	1.49	1.00–2.22	51.3	0.08
	Urinary system cancer	[17]	66	6 vs. 29	5 vs. 26	1.08	0.29–3.95	NA	NA
	Total		596	120 vs. 198	89 vs. 189	1.45	0.99-2.12	40.8	0.13
T category (T3 + 4 vs. T1 + 2)	Tumor type								
	Digestive system cancer	[15, 19]	238	96 vs. 24	95 vs. 23	0.98	0.46-2.08	25.1	0.26
	Urinary system cancer	[17]	66	22 vs. 13	8 vs. 23	4.87	1.69–14.0	NA	NA
	Head and neck cancer	[18]	190	44 vs. 73	16 vs. 57	2.15	1.10–4.19	NA	NA
	Total		494	162 vs. 110	119 vs. 103	1.68	0.82–3.43	60.8	0.04
N category (N^+^ vs. N^−^)	Tumor type								
	Digestive system cancer	[15, 19, 23]	465	164 vs. 119	91 vs. 91	1.02	0.53-1.93	58.5	0.05
	Head and neck cancer	[20, 21]	114	19 vs. 36	11 vs. 48	3.39	0.59-19.53	55.3	0.14
	Respiratory system cancer	[22]	124	44 vs. 59	11 vs. 10	0.68	0.26–1.74	NA	NA
	Population								
	Asian	[15, 19–21, 23]	579	183 vs. 155	102 vs. 139	1.26	0.68–2.33	58.8	0.02
	European	[22]	124	44 vs. 59	11 vs. 10	0.68	0.26–1.74	NA	NA
	Total		703	227 vs. 214	113 vs. 149	1.15	0.67–1.68	55.4	0.03
M category (M^+^ vs. M^−^)	Tumor type								
	Urinary system tumor	[17]	66	22 vs. 13	8 vs. 23	4.87	1.69–14.0	NA	NA
	Head and neck cancer	[18]	190	10 vs. 107	4 vs. 69	1.61	0.49–5.34	NA	NA
	Digestive system cancer	[19, 23]	213	4 vs. 117	4 vs. 88	0.72	0.09–5.83	54	0.14
	Total		469	36 vs. 237	16 vs. 180	2.51	1.20–5.26	46.2	0.10

*OR* odds ratio, *CI* confidence interval, *NA* not available

^a^The data of PDCD4 expression are expressed as the number of cases in the former subgroup versus the number of cases in the latter subgroup, e.g., the number of cases in the large tumor subgroup versus the number of cases in the small tumor subgroup

**Fig. 2:**
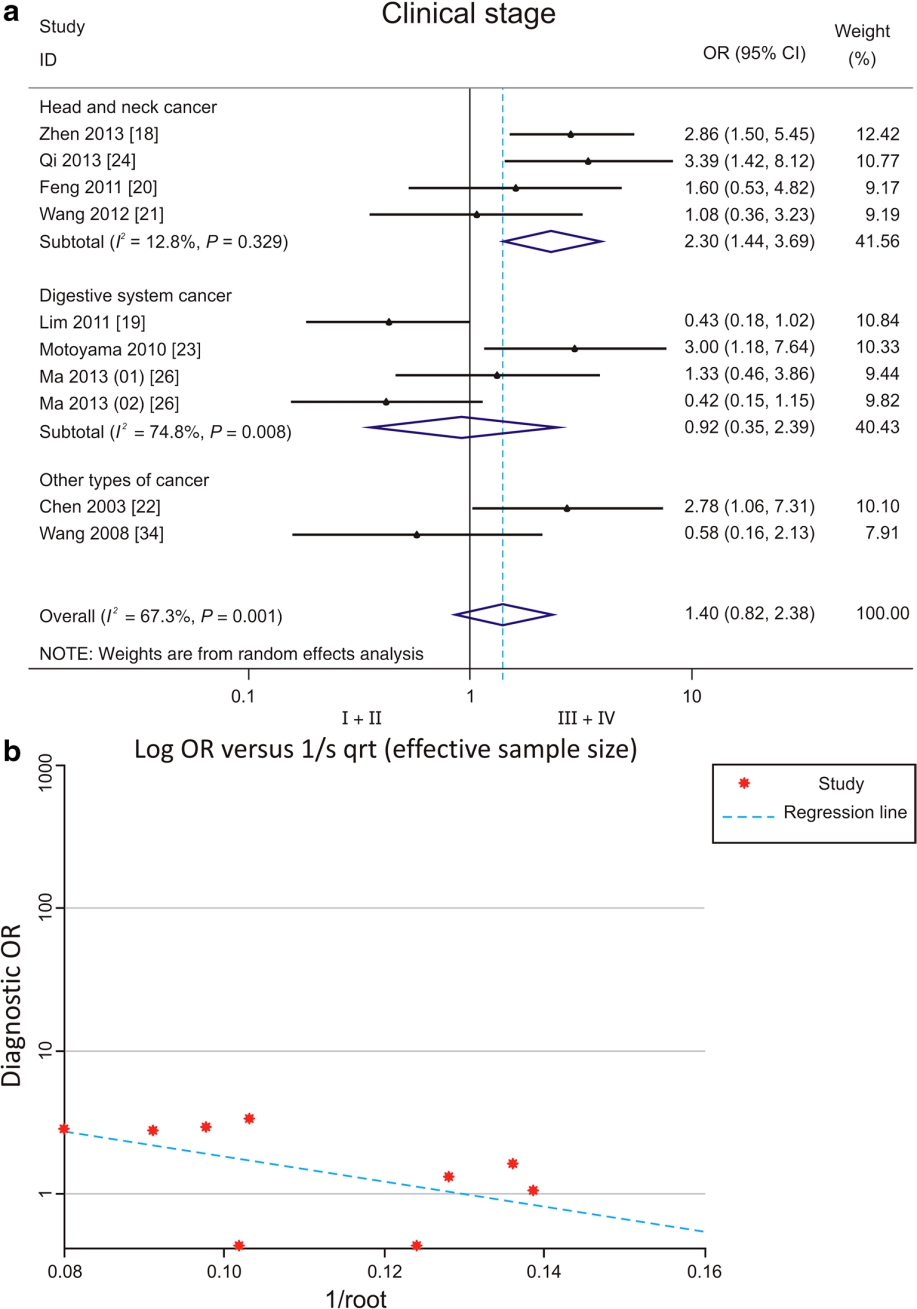
Forest plots and funnel plots demonstrating the associations between programmed cell death protein 4 (PDCD4) expression and clinical stage of various types of cancer. **a** Forest plot shows that low PDCD4 expression is significantly associated with advanced stages of head and neck cancers. Two cancer types were reported by Ma et al.: Ma [26] (01) for gastric cancer and Ma [26] (02) for pancreatic cancer. *OR* odds ratio, *CI* confidence interval. **b** Funnel plot shows no evidence of publication bias among papers on the association between PDCD4 expression and clinical stage of various types of cancer

**Fig. 3:**
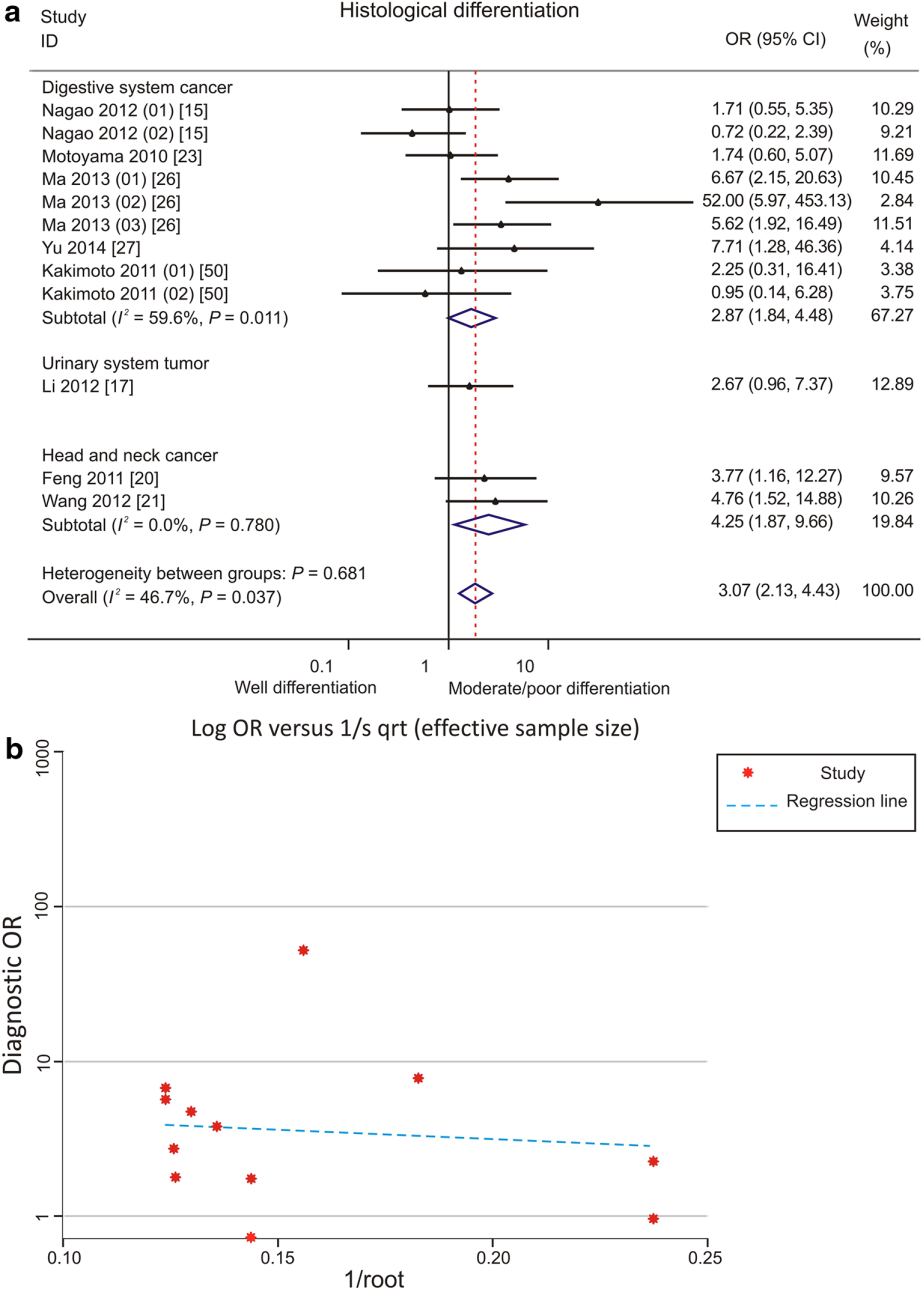
Forest plots and funnel plots demonstrating the associations between PDCD4 expression and histological differentiation of various types of cancer. **a** Forest plot shows that low PDCD4 expression is significantly associated with moderate/poor differentiation of digestive system cancers and head and neck cancers. Two sites of PDCD4 expression were reported by Kakimoto et al.: Kakimoto [50] (01) for PDCD4 expression in nucleus and Kakimoto [50] (02) for PDCD4 expression in cytoplasm of gastric cancer cells. Two sites of PDCD4 expression were reported by Nagao et al.: Nagao [15] (01) for PDCD4 expression in nucleus and Nagao [15] (02) for PDCD4 expression in cytoplasm of pancreatic cancer cells. Three cancer types were reported by Ma et al.: Ma [26] (01) for gastric cancer, Ma [26] (02) for pancreatic cancer, and Ma [26] (03) for colorectal cancer. *OR* odds ratio, *CI* confidence interval. **b** Funnel plot shows no evidence of publication bias among papers on the association between PDCD4 expression and histological differentiation of various types of cancer

Low PDCD4 expression was significantly associated with advanced stages of head and neck cancers (Fig. 2a). Low PDCD4 level was associated with moderately/poorly differentiated head and neck cancers (OR 4.25, 95% CI 1.87–9.66) [20, 21] and digestive system cancers (OR 2.87, 95% CI 1.84–4.48) [15, 23, 25–27] (Fig. 3a). No publication bias was observed (Figs. 2b, 3b).

### Quantitative analysis of the association between PDCD4 expression and OS

**Fig. 4:**
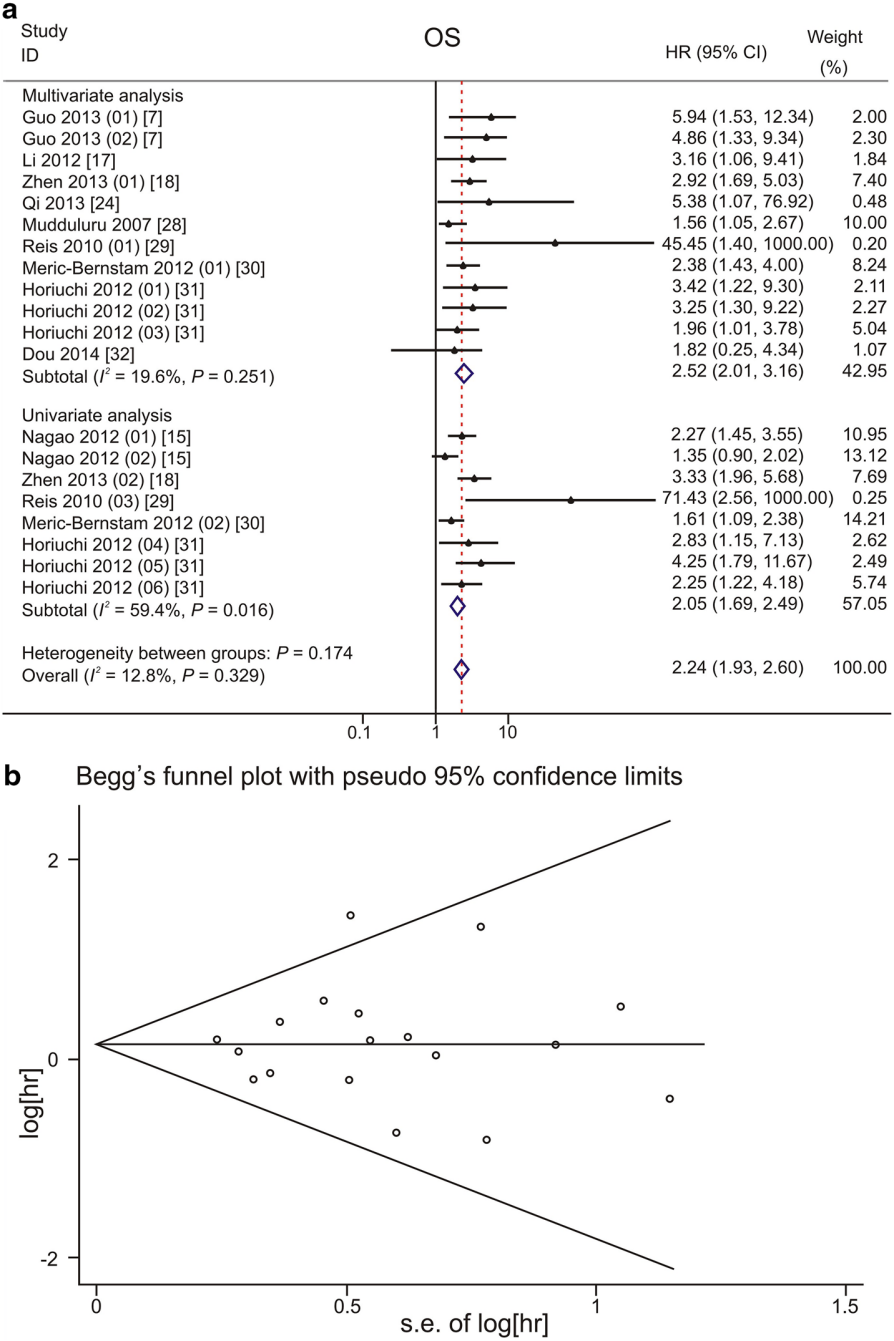
Forest plots and funnel plots demonstrating the associations between PDCD4 expression and overall survival (OS) of patients with solid tumors. **a** Forest plot shows that low PDCD4 expression level is significantly associated with a low OS rate. In total, 20 studies from 10 papers were included. Two T subcategories of gastric cancer were reported by Guo et al.: Guo [7] (01) for pT2a gastric cancer and Guo [7] (02) for pT2b gastric cancer. Two sites of PDCD4 expression were reported by Nagao et al.: Nagao [15] (01) for PDCD4 expression in nucleus and Nagao [15] (02) for PDCD4 expression in cytoplasm of pancreatic cancer cells. Different Duke’s stages were reported by Horiuchi et al.: Horiuchi [31] (01) for Duke’s stage B, Horiuchi [31] (02) for Duke’s stage C, and Horiuchi [31] (03) for Duke’s stage D colorectal cancer in multivariate analysis; Horiuchi [31] (04) for Duke’s stage B, Horiuchi [31] (05) for Duke’s stage C, and Horiuchi [31] (05) for Duke’s stage D colorectal cancer in univariate analysis. A fixed effects model was used to calculate the pooled hazard ratio (HR) for OS. Significant associations are observed in univariate model and in multivariate model. **b** Funnel plot shows no evidence of publication bias among papers on the association between PDCD4 expression and overall survival rate of various types of cancer. *s.e.* standard error. The *P* value is 0.940 in Begg’s test and is 0.744 in Egger’s test

Combined analysis of the 20 studies in 10 papers that addressed OS [7, 15, 17, 18, 24, 28–32] showed that reduced PDCD4 expression had an unfavorable impact on the OS of patients with solid tumors (HR: 2.24, 95% CI 1.93–2.60) (Table 3). Significant associations were observed in the univariate model (HR: 2.05, 95% CI 1.69–2.49) and multivariate model (HR: 2.52, 95% CI 2.01–3.16) (Fig. 4a). Subgroup analysis of different tumor types (Table 3) showed that reduced PDCD4 expression had an unfavorable impact on the OS of patients with head and neck cancers (HR: 3.24, 95% CI 1.93–5.45) [18, 24, 29], breast cancers (HR: 1.86, 95% CI 1.36–2.54) [30], digestive system cancers (HR: 2.12, 95% CI 1.75–2.56) [7, 15, 28, 31, 32], and urinary system cancers (HR: 3.16, 95% CI 1.06–9.41) [17]. No evidence of significant publication bias was observed with Begg’s test (*P* = 0.940) and Egger’s test (*P* = 0.744) (Fig. 4b).

**Table 3:** Subgroup analysis on the associations between PDCD4 expression and OS, DSS, and DFS/RFS of patients with solid tumors

Parameter	OS					DSS					DFS/RFS				
	Reference(s)	HR	95% CI	*I* ^*2*^ (%)	*P*	References	HR	95% CI	*I* ^*2*^ (%)	*P*	References	HR	95% CI	*I* ^*2*^ (%)	*P*
Total	[7, 15, 17, 18, 24, 28–31, 33]	2.24	1.93–2.60	42.1	0.025	[24, 28, 32, 34]	3.59	1.23–10.50	80.5	0.002	[29–31, 33, 35]	2.01	1.58–2.56	28.6	0.181
Univariate	[15, 18, 29–31]	2.05	1.69–2.49	59.4	0.016	None	NA	NA	NA	NA	[29–31]	2.05	1.16–3.63	51.1	0.105
Multivariate	[7, 17, 18, 24, 28–31, 33]	2.52	2.01–3.16	19.6	0.251	[24, 28, 32, 34]	3.59	1.23–10.50	80.5	0.002	[29–31, 33, 35]	2.37	1.69–3.33	0	0.456
Brain tumor	None	NA	NA	NA	NA	[32]	15.87	3.62–71.43	NA	NA	None	NA	NA	NA	NA
Univariate	None	NA	NA	NA	NA	None	NA	NA	NA	NA	None	NA	NA	NA	NA
Multivariate	None	NA	NA	NA	NA	[32]	15.87	3.62–71.43	NA	NA	None	NA	NA	NA	NA
Head and neck cancer	[18, 24, 29]	3.44	2.38–4.98	41.9	0.142	[24]	5.05	1.12–62.50	NA	NA	[29]	2.10	1.30–3.39	47.4	0.149
Univariate	[18, 29]	10.70	0.58–197.75	74.5	0.047	None	NA	NA	NA	NA	[29]	7.46	1.04–52.63	NA	NA
Multivariate	[18, 24, 29]	3.24	1.93–5.45	29.7	0.241	[24]	5.05	1.12–62.50	NA	NA	[29]	1.94	1.19–3.18	52.4	0.147
Breast cancer	[30]	1.86	1.36–2.54	28.1	0.238	None	NA	NA	NA	NA	[30]	1.23	0.79–1.92	NA	NA
Univariate	[30]	1.61	1.09–2.38	NA	NA	None	NA	NA	NA	NA	[30]	1.23	0.79–1.92	NA	NA
Multivariate	[30]	2.38	1.43–4.00	NA	NA	None	NA	NA	NA	NA	None	NA	NA	NA	NA
Digestive system cancer	[7, 15, 28, 31]	2.12	1.75–2.56	36.2	0.101	[28]	1.31	1.00–1.72	NA	NA	[31, 33]	2.57	1.74–3.78	0	0.877
Univariate	[15, 31]	2.14	1.49–3.06	43.8	0.130	None	NA	NA	NA	NA	[31]	2.50	1.45–4.30	0	0.857
Multivariate	[7, 15, 28, 31]	2.33	1.73–3.13	36.6	0.149	[28]	1.31	1.00–1.72	NA	NA	[31, 33]	2.64	1.52–4.58	0	0.562
Gynecologic tumor	None	NA	NA	NA	NA	[34]	3.36	1.43–7.81	NA	NA	[35]	3.42	1.41–8.33	NA	NA
Univariate	None	NA	NA	NA	NA	None	NA	NA	NA	NA	None	NA	NA	NA	NA
Multivariate	None	NA	NA	NA	NA	[34]	3.36	1.43–7.81	NA	NA	[35]	3.42	1.41–8.33	NA	NA
Urinary system cancer	[17]	3.16	1.06–9.41	NA	NA	None	NA	NA	NA	NA	None	NA	NA	NA	NA
Univariate	None	NA	NA	NA	NA	None	NA	NA	NA	NA	None	NA	NA	NA	NA
Multivariate	[17]	3.16	1.06–9.41	NA	NA	None	NA	NA	NA	NA	None	NA	NA	NA	NA
Asian	[7, 15, 17, 18, 24, 28, 31]	2.46	2.06–2.95	23.5	0.193	[24, 32, 34]	5.67	2.13–5.12	36.6	0.206	[31, 33, 35]	2.69	1.88–3.83	0	0.908
Univariate	[15, 18, 31]	2.34	1.66-3.30	50.6	0.072	None	NA	NA	NA	NA	[31]	2.50	1.45–4.30	0	0.857
Multivariate	[7, 17, 24, 28, 31]	3.05	2.26–4.11	0.0	0.750	[24, 32, 34]	5.67	2.13–5.12	36.6	0.206	[31, 33, 35]	2.84	1.78–4.53	0	0.708
European	[28]	1.56	1.05–2.67	NA	NA	[28]	1.31	1.00–1.72	NA	NA	None	NA	NA	NA	NA
Univariate	None	NA	NA	NA	NA	None	NA	NA	NA	NA	None	NA	NA	NA	NA
Multivariate	[28]	1.56	1.05–2.67	NA	NA	[28]	1.31	1.00–1.72	NA	NA	None	NA	NA	NA	NA
North American	[29, 30]	1.99	1.46–2.71	71.7	0.014	None	NA	NA	NA	NA	[29, 30]	1.58	1.14–2.18	52.9	0.095
Univariate	[29, 30]	7.95	0.20–311.36	83.6	0.014	None	NA	NA	NA	NA	[29, 30]	2.33	0.43–12.57	67.5	0.080
Multivariate	[29]	2.56	1.54–4.25	66.9	0.082	None	NA	NA	NA	NA	[29]	1.94	1.19–3.18	52.4	0.147
Publication bias															
Begg’s test					0.940					0.308					0.902
Egger’s test					0.744					0.215					0.550

*OS* overall survival, *DSS* disease-specific survival, *DFS* disease-free survival, *RFS* relapse-free survival, *HR* hazard ratio, *CI* confidence interval, *NA* not available

### Quantitative analysis of the association between PDCD4 expression and DSS

**Fig. 5:**
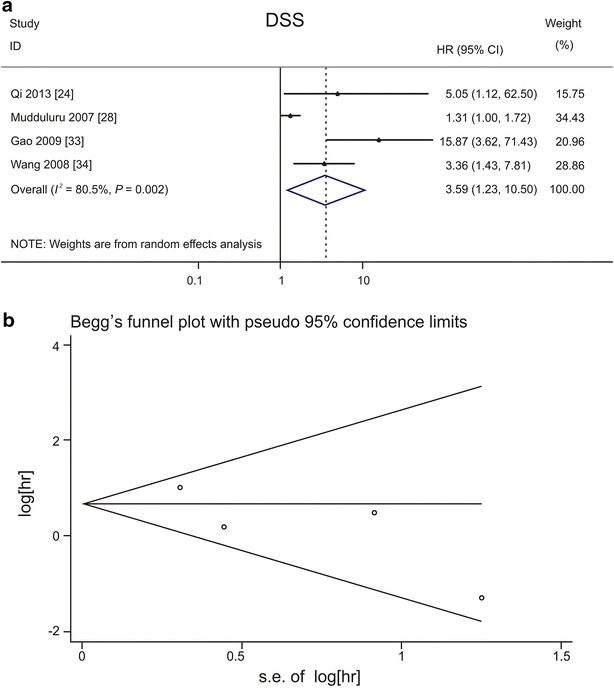
Forest plots and funnel plots demonstrating the associations between PDCD4 expression and disease-specific survival (DSS) of patients with solid tumors. **a** Forest plot shows that low PDCD4 expression level is significantly associated with a low DSS rate. A random effects model was used to calculate the pooled HR for DSS. **b** Funnel plot shows no evidence of publication bias among papers on the association between PDCD4 expression and disease-specific survival rate of various types of cancer. The *P* value is 0.308 in Begg’s test and is 0.215 in Egger’s test

In total, 4 studies that addressed DSS were pooled for analysis [24, 28, 33, 34]. The pooled HR showed significant differences in DSS between the high and low PDCD4 expression groups (HR: 3.59, 95% CI 1.23–10.50) (Fig. 5a). Down-regulation of PDCD4 expression was associated with unsatisfactory DSS in patients with head and neck cancers (HR: 5.05, 95% CI 1.12–62.50) [24], brain tumors (HR: 15.87, 95% CI 3.62–71.43) [33], and gynecologic cancers (HR: 3.36, 95% CI 1.43–7.81) [34]. For digestive system cancers, no association was demonstrated (HR: 1.31, 95% CI 1.00–1.72) [28]. An association between low PDCD4 expression and short DSS was found in patients from Asia (HR: 5.67, 95% CI 2.13–5.12) but not in patients from Europe (HR: 1.31, 95% CI 1.00–1.72) (Table 3). The Begg’s test (*P* = 0.308) and Egger’s test (*P* = 0.215) showed no significant publication bias (Fig. 5b).

### Quantitative analysis of the association between PDCD4 expression and DFS/RFS

**Fig. 6:**
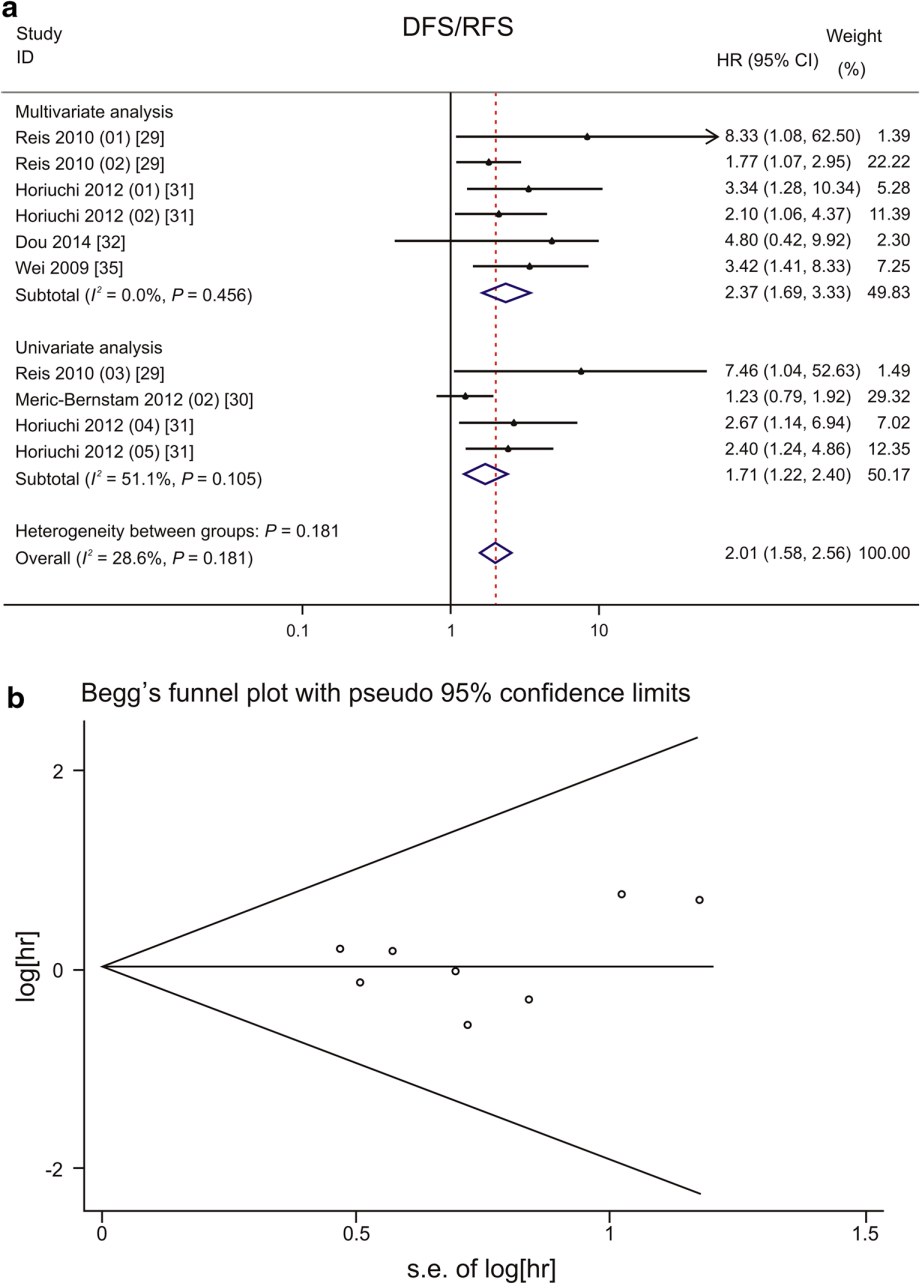
Forest plots and funnel plots demonstrating the associations between PDCD4 expression and disease-free survival (DFS)/relapse-free survival (RFS) of patients with solid tumors. **a** Forest plot shows that low PDCD4 expression level is significantly associated with a low DFS/RFS rate. In total, 10 studies in 5 papers were included. A fixed effects model was used to calculate the pooled HR for DFS/RFS. Significant associations are observed in univariate model and in multivariate model. Different Duke’s stages were reported by Horiuchi et al.: Horiuchi [31] (01) for Duke’s stage B and Horiuchi [31] (02) for Duke’s stage C colorectal cancer in multivariate analysis; Horiuchi [31] (04) for Duke’s stage B and Horiuchi [31] (05) for Duke’s stage C colorectal cancer in univariate analysis. **b** Funnel plot shows no evidence of publication bias among papers on the association between PDCD4 expression and disease-free survival rate of various types of cancer. The *P* value is 0.902 in Begg’s test and is 0.550 in Egger’s test

The pooled HR for DFS/RFS showed a significant association with PDCD4 down-regulation (HR: 2.01, 95% CI 1.58–2.56) (Fig. 6a) [29–32, 35]. Low PDCD4 expression was associated with short DFS/RFS of patients with head and neck cancers (HR: 2.10, 95% CI 1.30–3.39) [29], digestive system cancers (HR: 2.57, 95% CI 1.74–3.78) [31, 32], and gynecologic cancers (HR: 3.42, 95% CI 1.41–8.33) [35]; for breast cancer patients, no association was observed (HR: 1.23, 95% CI 0.79–1.92) [30] (Table 3). The Begg’s test (*P* = 0.902) and Egger’s test (*P* = 0.550) showed no significant publication bias (Fig. 6b).

## Discussion

In this meta-analysis, we examined the association between PDCD4 expression and the clinicopathologic parameters of cancers from different anatomical sites, including the brain, head and neck, breast, and digestive, gynecologic, and urinary systems. Our results indicated a strong negative association of PDCD4 expression with tumor size and differentiation status of solid tumors. In a stratified analysis, reduced PDCD4 expression level was associated with late T subcategories in head and neck tumors and urinary system cancers as well as distant metastasis of urinary system cancers. Furthermore, low PDCD4 expression was associated with advanced stage head and neck cancers and respiratory system cancers.

Epithelial differentiation was a determining factor in the prognoses of head and neck cancers. Poorly differentiated cancers were highly proliferative compared with their highly differentiated counterparts. In addition, differentiation status was important in maintaining a tumorigenic and treatment-resistant cancer stem cell subpopulation in head and neck cancers [36]. Hence, cancer treatment with differentiation inducers (such as retinoic acid) could inhibit cancer cell proliferation and is known as differentiation cancer therapy [37, 38]. In head and neck cancers, it had been demonstrated that valproic acid or all-trans retinoic acid could inhibit the growth of head and neck squamous cell carcinomas by inducing terminal differentiation [39]. Furthermore, differentiation therapy could help in suppressing and eradicating the cancer stem cell population in head and neck squamous cell carcinomas [36, 40]. The association between PDCD4 expression and cancer cell differentiation was clearly demonstrated in peripheral blood cancers. PDCD4 expression was induced in NB4 and HL-60 acute myelocytic leukemia (AML) cell lines, primary human promyelocytic leukemia (AML-M3) cells, and CD34^+^ hematopoietic progenitor cells in the presence of all-trans retinoic acid. Differentiation induction could be prevented if PDCD4 was silenced using small interfering RNA (siRNA) [41]. In solid tumors, PDCD4 expression was inhibited by the phosphatidylinositol 3-kinase (PI3K)/protein kinase B (PKB/AKT)/mammalian target of rapamycin (mTOR) pathway. During adipogenic differentiation of adipose tissue-derived mesenchymal stem cells, PDCD4 expression was reduced and AKT phosphorylation was increased in a time-dependent manner [42]. Because of the association between PDCD4 and cancer differentiation, PDCD4 restoration could be a novel approach for differentiation cancer therapy, resulting in effective suppression of solid tumor development and further improving the prognosis [43].

In urinary system cancer, PDCD4 suppression was associated with the metastatic status of the patients [17]. PDCD4-knockout mice developed spontaneous lymphomas with systematic dissemination and frequent liver/renal metastasis [44]. Preclinical studies have indicated that PDCD4 could control key genes involved in cancer migration and metastasis [25, 45, 46]. The expression of urokinase plasminogen activator surface receptor (u-PAR), which mediates plasmin-mediated extracellular matrix degradation, was shown to be controlled by PDCD4 [45]. In PDCD4-knockdown cancer cells, epithelial cadherin 1 (E-cadherin) promoter activity was inhibited. In contrast, in colorectal cancer cells overexpressing PDCD4, E-cadherin protein level was increased accordingly [25]. In ACHN and 786-O renal cancer cells, PDCD4 regulated AKT phosphorylation, leading to the migration or invasion of cancer cells via the up-regulation of the mammalian target of rapamycin complex 1 (mTORC1) [46]. Metastasis of cancer cells is a main cause of death in laryngeal carcinoma patients. Moreover, molecular factors involved in metastasis, especially in the epithelial-mesenchymal transition (EMT), could be a possible mechanism of cancer cell resistance. Because low PDCD4 expression was significantly associated with metastasis in urinary system cancers, PDCD4 could be a novel target for improving the prognosis of this malignancy.

Several limitations were observed in this meta-analysis. A small sample size was observed in one cancer group. Moreover, multiple cut-off criteria, discrepancies among diverse tumor properties, and the therapy received also affected the results. In most of the studies included in prognostic assessment, all clinical stages were represented in the respective cancer cases. However, the study reported by Dou et al. [32], only focused on advanced stage rectal cancer, and the low PDCD4 expression within these patients was not significantly associated with the 5-year OS or DFS indicated by the 95% CI overlapping 1. Furthermore, the subcellular localization of the PDCD4 protein affects cancer behavior. During tumor progression, PDCD4 protein translocation from the nucleus to the cytoplasm was observed in cancer cells [47, 48]. Accumulation of cytoplasmic PDCD4 protein is reported in both normal and cancer cell lines [49]. Almost all of the studies included in our meta-analysis measured the total PDCD4 protein level in the tissue samples rather than the separate nuclear or cytoplasmic PDCD4 protein level. In the study conducted by Nagao et al. [15], both nuclear and cytoplasmic PDCD4 protein pools were examined in pancreatic cancer patients. Different ORs and 95% CIs were obtained for the assessment of clinical stage and OS. A similar situation was observed in the study by Kakimoto et al. [50] that examined the association between PDCD4 expression and histological differentiation. These findings suggest that the prognostic value of PDCD4 in human malignancies should be studied by further stratified analysis, including the determination of precise cellular localization of PDCD4.

## Conclusions

Our results demonstrated that reduced expression of PDCD4 in solid tumors is an unfavorable prognostic indicator. We noticed that the sample size for a particular cancer group was not large enough to define the prognostic value of PDCD4, and not having nucleus-specific PDCD4 protein measurements could also restrict the precise evaluation. Future studies on a larger scale are warranted to address the association of PDCD4 with the unique clinical features presented by different cancers.
